# Review on various activator-assisted polymer grafting techniques for smart drug delivery applications

**DOI:** 10.1039/d5ra03188e

**Published:** 2025-07-04

**Authors:** Rahul Bera, Reechik Bandyopadhyay, Biplab Debnath, Gouranga Dutta, Abimanyu Sugumaran

**Affiliations:** a Department of Industrial Pharmacy, Bharat Technology Uluberia Howrah 711316 India gourangadutta071994@gmail.com; b Department of Pharmaceutical Chemistry, Bharat Technology Uluberia Howrah 711316 India; c Department of Pharmaceutical Sciences, Sushruta School of Medical and Paramedical Sciences, Assam University (A Central University) Silchar 788011 Assam India abipharmastar@gmail.com abimanyu.s@aus.ac.in

## Abstract

Activator-assisted polymer grafting has emerged as a crucial approach in the development of advanced drug delivery systems, enabling precise regulation of drug release, targeting, and biocompatibility. In contrast to traditional formulation techniques, activator-mediated grafting methods, including redox initiation, photo-induced reactions, plasma treatment, and enzymatic catalysis, provide improved functionalization of polymers, achieving high grafting efficiency and adjustable properties. This review presents a thorough assessment of polymer grafting methodologies, encompassing grafting-to, grafting-from, and grafting-through techniques, with a detailed examination of the function of activators in facilitating these processes. The focus is directed towards enhancing polymer solubility, mucoadhesion, and responsiveness to physiological stimuli through these strategies, ultimately leading to optimized therapeutic performance. Furthermore, the review examines current developments and biomedical applications of activator-assisted grafted polymers, particularly in targeted drug delivery, tissue engineering, and formulations tailored for specific diseases. The insights presented aim to guide the development of advanced polymers that demonstrate superior efficacy, minimize systemic toxicity, and enhance patient adherence.

## Abstract 2

Activator-assisted polymer grafting has emerged as a crucial approach in the development of advanced drug delivery systems, enabling precise regulation of drug release, targeting, and biocompatibility.

## Graphical abstract

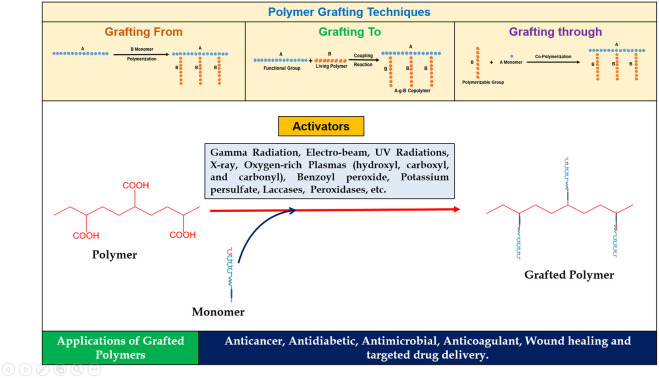

## 1. Introduction

The constant evolution of disease patterns and types has made targeted drug delivery a growing challenge in the modern era. Greater attention is necessary for the treatment of lifestyle diseases and chronic, sub-chronic conditions. These diseases require complex and sophisticated delivery systems for excipients with distinctive properties, which allow them to target specific areas while controlling drug release and maintaining a safe drug release profile.^1^ Numerous researchers have design a variety of innovative techniques to improve biocompatibility, targeted drug delivery, and controlled, sustainable release in order to satisfy the current demands. One of the most prevalent and contemporary techniques is the modification of excipients, such as polymers.^2^ These base excipients are a formulation's fundamental, pharmacologically inert components that facilitate the preparation, delivery, and stability of a drug. Passive carriers can be converted into effective delivery vehicles through modification, particularly chemical grafting.

Polymers are classified as homopolymers, copolymers, or block copolymers based on their monomer composition. Copolymers consist of two or more monomer units, and their names usually indicate the arrangement (*e.g.*, random, alternating, block) and origin of these monomers, significantly affecting their chemical reactivity and grafting behavior.

Polymers are capable of forming structures that can range from simple films to complex nanoparticles, and those can be derived from natural or synthetic sources. Polymers are the fundamental components of almost every biomedical device and formulation. Their properties depend on their chemical structures and sources. Nevertheless, unmodified and simple polymers frequently encounter obstacles, including weak adhesion, poor solubility, and restricted targeting capabilities. In general, biocompatibility, stability, biodegradability, ability to regulate drug release, and efficacy in reaching the intended site are the factors used to evaluate a “good” drug delivery system. Polymer-based systems are capable of satisfying these criteria as a result of their modifiability and adjustable degradation rates. However, these also encounter many limits. It is one of the conceivable methods to counter these polymer structural modifications. The polymer grafting process can be conducted on the backbone of the polymer chain. These will improve the therapeutic efficacy, controlled release, and site-specificity of the polymer by enhancing its interaction with biological environments.^3,4^

Polymer grafting is a key strategy for improving drug delivery systems. Grafting alters the physicochemical properties and biological performance of base polymers by chemically attaching functional side chains to their backbone. This method enables the creation of specialized polymers tailored to specific pharmaceutical objectives, including enhanced solubility, wettability, water retention, and morphology. Grafted polymers can be designed to respond to physiological stimuli such as pH, temperature, ionic strength, redox conditions, or enzymatic activity, allowing for site-specific and controlled drug release.^5,6^ Several hybrid nanobiomedical platforms that integrate with grafted polymers have become increasingly popular in order to enhance drug delivery and therapeutic applications. In particular, metal–organic frameworks (MOFs) are porous crystalline materials composed of metal ions and organic moieties, offering advantages like surface functionalization, tunable pore diameters, and high drug-loading capacity, controlled or targeted drug release. MOF-polymer hybrids are a next-generation drug delivery strategy that provides synergistic benefits in terms of both precision and efficacy when combined with polymer grafting.^7–10^

This polymer grafting technique has been started in the 1980s for modifying the polymers to improve their physicochemical properties.^11^ The polymer grafting involves three major methods: grafting-to, grafting-from, and grafting-through.^12^ Numerous investigations have been conducted extensively on generic polymer grafting mechanisms and their applicability across diverse sectors.^12,13^ Several studies have concentrated on distinct grafting strategies applied for various forms of chain alteration on demand for various applications.^14,15^ Remarkably, only a limited number of studies have focused on the activators linked to polymer grafting processes. Activators play a crucial role in ensuring effective and successful grafting in polymers. This review seeks to fill this gap by offering a comprehensive and structured examination of activator-assisted polymer grafting techniques across various disease areas. While numerous publications have addressed conventional polymer grafting or specific initiator systems, there is a scarcity of thorough investigations into activator-assisted techniques and their impacts across therapeutic areas. This review distinctly gathers insights on polymer grafting, focusing on reaction types, biomedical applications, and efficiency, particularly as influenced by different classes of activators. This focus on areas such as cancer, diabetes, wound healing, and infectious disease treatment highlights new connections between activator chemistry and the effectiveness of drug delivery.

## 2. Overview of polymer brush and methodology

Polymer brushes are one way of representing the attachment of various chemical moieties to a polymer chain surface. These brushes are thin, brush-like structures with one end attached to a polymer chain and the other end extending outward. These chains are chemically anchored to the surface of polymer chains. This idea originated in the 1980s. These additions made those polymers more effective and demonstrated extraordinary qualities by altering their properties such as wettability, biocompatibility, friction, tissue adhesion, *etc*.^16^ Owing to their elevated grafting density, polymer brushes exhibit behaviors that are distinct from those of free polymers in solution, thereby providing unique advantages in applications such as antifouling coatings, drug delivery, and responsive surfaces. Specific polymer brushes are stimuli–responsive, exhibiting expansion or contraction in response to variations in pH, temperature, or ionic strength, rendering them versatile for applications in the fields of medicine, nanotechnology, and surface engineering. Polymer brushes are typically synthesized utilizing two principal grafting methodologies: grafting-to and grafting-from.

**Fig. 1:**
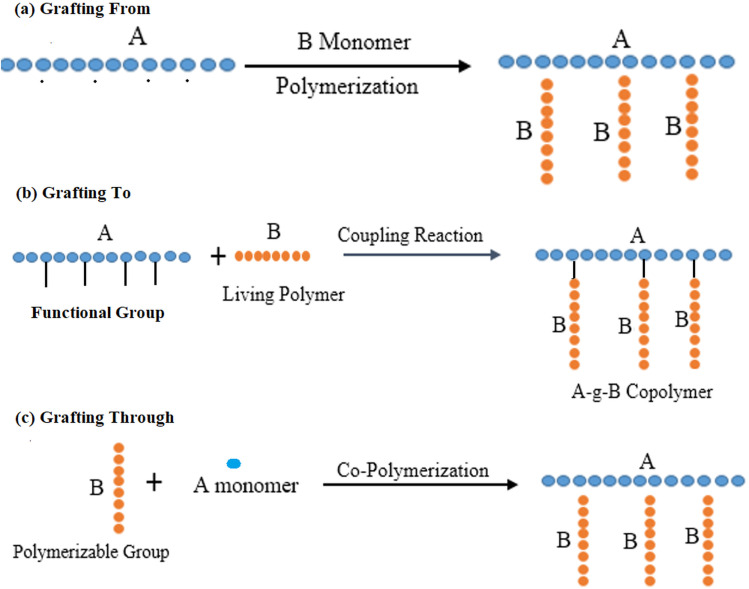
(a) ‘Grafting From’ technique: a conducting polymer core synthesized with initiation side functions as a macro-initiator from where side chains are grown (b) ‘Grafting To’ technique-pre-polymerized chains are attached to reactive core polymers; (c) ‘Grafting Through’ technique: after polymerization, macromonomers are synthesized to form core polymer.

Polymer grafting occurs through three principal methodologies (Fig. 1): (1) “grafting-to, where a polymer with a reactive end group bonds to functional groups located on the polymer's main chain; (2) grafting-from, wherein polymer chains are synthesized from initiator sites on the polymer's backbone; and (3) grafting-through, which involves the combination of a reactive macromolecule with a small-molecular-weight monomer”. Among these methodologies, the grafting-to and grafting-from techniques are the most widely utilized. The grafting-to method yields precisely defined graft segments, as polymerization occurs independently before the attachment of polymer chains to the primary backbone. Conversely, the grafting-from approach enhances the synthesis of compounds with elevated grafting densities by mitigating the steric barriers associated with the grafting-to method.^17^ Each polymer grafting technique has distinct advantages and disadvantages concerning the chemical composition, length, density, dispersity of the resulting graft, and the ease and efficacy of the associated chemical processes.^18^

The selection of grafting technique influences both graft density and architecture, while also profoundly affecting the operational efficacy of drug delivery systems. A grafting method utilizing rational molecular design enables the establishment or prediction of modifications to graft length, density, functional groups, and polymer backbones. This will enhance solubility, drug loading efficiency, targeting specificity, release kinetics, and biodistribution of the loaded medication.^19^ By optimizing the polymer backbone and graft length *via* molecular design, the drug loading capacity can be augmented through improved interaction sites or encapsulation domains. The incorporation of targeting ligands, such as surface grafting with biological ligands (*e.g.*, antibodies, peptides, folic acid), facilitates receptor-mediated targeting and absorption by binding to overexpressed receptors. Stimuli–responsive grafts, responsive to pH, temperature, or enzymes, provide regulated or on-demand release patterns under certain physiological circumstances.^20^ Moreover, including biodegradable components ensures the secure disintegration and removal of the delivery system after therapy, thereby mitigating long-term toxicity. These design factors jointly optimize biodistribution, improve therapeutic accuracy, and boost overall therapy efficacy.^12^

The chemistry of polymer grafting is primarily driven by the reactivity of the various functional groups found in the polymer backbone and monomers. These functional groups include hydroxyl (–OH), carboxyl (–COOH), amine (–NH_2_), thiol (–SH), *etc.* During the grafting reactions, reactive macroradicals are generated on either the polymer backbone or the monomer by various activators, such as physical, chemical, biological, or combined. These macroradicals subsequently migrate through chain-growth mechanisms. The rate of reaction, selectivity, and efficiency of grafting are directly influenced by the nature of the activators, which may also influence variations in polymer architecture and side reactions.

The polymer grafting procedures involve several steps for the successful preparation of grafted polymers. Following preparation, the products are collected; therefore, assuring the purity of the grafted polymer is essential for maintaining quality and safety in biological applications.^13^ The standard chemical synthesis methods were utilized to purify grafted polymers, which included dialysis, solvent precipitation, and ultrafiltration, to eliminate residual components, unreacted precursors, and by-products. These processes are necessary for assuring the safety of the synthesized product.^21^ This assessment will ensure the potential toxicity from the monomers or the byproducts. These purification techniques are essential for making a safe and biocompatible newly grafted polymer.

However, evaluating the synthesized grafted polymer is necessary to understand its structural orientation and confirm successful grafting. Additional characterizations help to assess the characteristics of the newly grafted polymer, which are essential for biological applications.^13,22^ The widely used characterization techniques, such as ^1^H Nuclear Magnetic Resonance (NMR) and ^13^C NMR, as well as Infrared Spectroscopy (IR), which are extensively utilized to identify grafting sites, confirm chemical structures, and detect specific functional groups introduced through the grafting of modified polymers. The microscopic assessment provides insight into the surface morphology. X-ray diffraction (XRD) analysis reveals the alterations in crystallinity of the polymer. In contrast, Differential Scanning Calorimetry (DSC) and Thermogravimetric Analysis (TGA) assess the thermal behavior and stability profile of the grafted polymer. Size Exclusion Chromatography (SEC) is utilized to ascertain the average molecular weights and distributions of grafted chains. Collectively, these methodologies yield a thorough physicochemical characterization of grafted polymers, facilitating enhanced connection between structural attributes and functional efficacy. However, beyond analytical accuracy, these methods are also essential for removing unreacted monomers, initiators, or side products that could compromise biocompatibility. This characterization is essential for guaranteeing repeatability, batch quality control, and regulatory approval for biological applications.^22^

## 3. Activators in polymer grafting

**Fig. 2:**
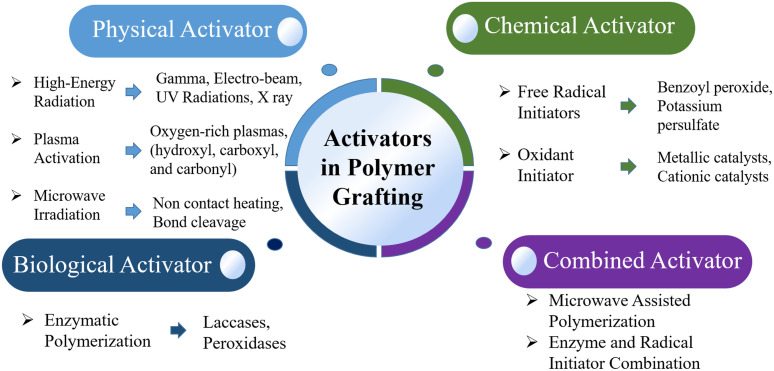
Various activators and their examples for polymer grafting techniques.

Activators play a crucial role in polymer grafting, initiating the process by creating reactive sites on the polymer backbone that allow for the attachment of new chains or functional groups. The choice of activator has a significant impact on the efficiency, specificity, and properties of the grafted polymer. Broadly categorized as physical, chemical, or biological, each type of activator offers unique advantages. Using specific or combined activators enables highly customized grafting and tailoring of material properties for diverse applications, such as biomedical devices, environmental materials, and advanced coatings (Fig. 2).

### 3.1. Physical activators

Physical activators are mostly external high-energy radiations to chemical reactions. Physical activators include gamma, plasma, and microwave radiation. These high-energy radiations created free and excited ions in the reaction chamber and caused polymer chains to interact with the added chemical. Localized and controlled activators allow seamless polymer chain grafting.

#### 3.1.1 High-energy radiation

**Fig. 3:**
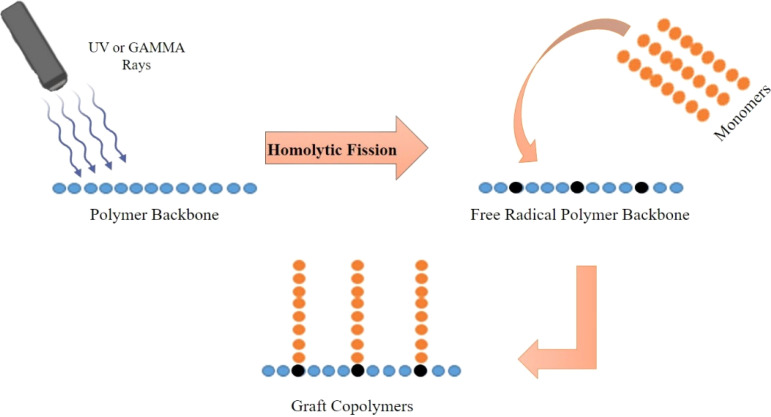
Schematic diagram of high energy radiation activation of polymer backbone for polymer grafting.

High-energy radiation includes gamma rays, electrons, X-rays, and other forms of radiation. This is highly effective for the grafting techniques. This category contains three grafting procedures. In pre-irradiation, high-energy beams activate polymers, creating macroradicals. These polymer-bound radicals, formed during activation, act as reactive sites for grafting. Grafting polymerizations proceed when these macroradicals react with monomer molecules^23^ (Fig. 3). The reaction initiated in an oxygen environment can cause the generation of highly reactive oxygen species, which react with the polymer chains, cleaving the polymer to form radicals. These highly reactive polymer radicals react with the monomers.^24^ These procedures are clean and catalyst-free. These can scale for biological applications using thermally sensitive polymers.^25^

#### 3.1.2 Plasma activation

Plasma activators enable polymer surface grafting for numerous objectives. Plasma is an ionized gas composed of oxygen, nitrogen, and argon. Ionized gases react with polymer surfaces to graft chemical moieties in two ways. Plasma may generate free radicals on the polymer surface that might attract monomers or chemical moieties. Plasma sometimes adds polar functional groups like –OH, –COOH, and –NH_2_ to polymers. External monomers connect to these functional groups.^26–28^ These methods are precise and successful for surface grafting. Adjusting power, treatment duration, and gas concentration controls response rates.

#### 3.1.3 Microwave activators

Another high-energy grafting activator is microwave radiation. Rapid internal heating from microwave radiation starts chemical processes. This radiation quickly created polymer free radicals that interacted with chemical moieties. These activators react quickly and require less power. These polymer grafting methods are eco-friendly. Microwave-activated polymers exhibit enhanced mechanical and chemical properties, rendering them suitable for the development of biological and advanced materials.^29,30^

### 3.2. Chemical activators

Chemical activators are substances that directly or indirectly generate reactive species, such as radicals or non-radicals, reactive ions, *etc.*, which facilitate the grafting process through the reaction between the polymer backbone and the monomers. Some chemical activation techniques are used for polymer grafting, such as free radical activation, redox-generated activation, and click chemistry.

#### 3.2.1 Free radical activators

Free radical initiators are a popular method for polymer grafting. A free radical is a small, highly reactive molecule (*e.g.*, hydroxyl or alkyl radicals), which enables high efficiency and compatibility with a wide range of polymer grafts. They initiated the grafting process through the generation of free radicals on the polymer backbone, creating a reactive site for anchoring the monomers and extending the polymer chains. There are a few chemical initiators used for grafting reactions, such as “benzoyl peroxide (BPO), azobisisobutyronitrile (AIBN), *tert*-butyl peroxide, potassium persulfate (K_2_S_2_O_8_), ammonium persulfate (APS), *etc*”. These initiators are cleaved at certain temperatures and made highly reactive free radicals. The key factors for these processes are controlled temperatures and concentrations of these initiators.^12,31^

#### 3.2.2 Other chemical activators

In addition to the free radical initiators, other chemical initiators are present during polymer anastomosis. Redox initiators are one of the chemical activation techniques. In this process, a limited number of chemicals (*e.g.*, Fenton's reagent, ceric ammonium nitrate) are employed to establish a redox system that produces free radicals from reactive compounds, facilitating grafting. This reduces the likelihood of thermal degradation, as these systems are more controlled and can be executed at lower temperatures. The grafting process is initiated by the electron-transferring redox system.^32,33^ The most prevalent click reactions utilized in polymerization and modification encompass azide–alkyne cycloaddition (AAC), Diels–Alder reactions, thiol-X reactions, and carbonyl-based additions, among others. The primary reaction employed in click chemistry systems is the copper(i)-catalyzed azide–alkyne cycloaddition (CuAAC). The system progressed through several stages, resulting in the generation of Cu(l) from CuSO_4_, followed by the reaction of terminal alkynes with azides utilizing reducing agents. These reactions exhibit high selectivity and are among the most effective methods for modifying and functionalizing polymer surfaces. Thiol–ene and thiol–yne reactions, along with other click chemistry systems, are utilized as well. These reactions are advantageous because of their high specificity, reproducibility, and low by-product formation. These strategies facilitate site-specific grafting of pharmaceuticals, imaging agents, or ligands through post-polymerization modification. This enables accurate regulation of the grafting site and density of the grafting polymer, in addition to controlling release profiles and targeting functionalities.^34,35^

### 3.3. Biological activators

**Fig. 4:**
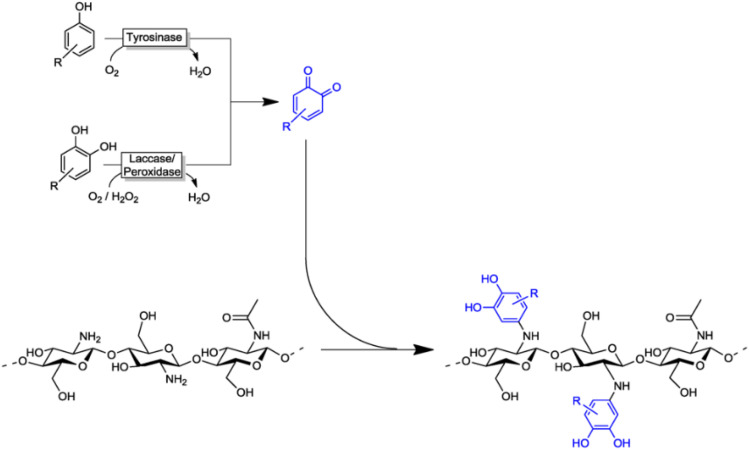
The Laccase enzyme activators mediated chitosan grafting of quinones. (Figure adapted from: “Enzyme Initiated Radical Polymerizations” by Hollmann *et al.*,^43^ licensed under CC BY 3.0. https://www.creativecommons.org/licenses/by/3.0/.)

Biological activators are one of the additional activators used in polymer grafting. Polymer grafting is predominantly facilitated by enzymes, which catalyze reactions under moderate, environmentally favorable conditions. These methods are advantageous for grafting natural polymers or biopolymers, as they enable the particular and non-destructive modification of polymer chains. Enzymatic polymerization is an advanced method of polymer grafting. In this process, enzymes act as catalysts to facilitate the grafting of natural polymers with different monomers. This offers advantages such as high specificity, mild reaction conditions, and minimal polymer backbone degradation. The most commonly used enzyme in this technique is called laccase. Laccase oxidizes phenolic compounds in polymers to generate phenoxy radicals, which form covalent bonds with monomers, modifying the polymer structure. This controlled grafting process reduces side reactions and preserves polymer integrity. That is crucial for natural polymers to maintain their integrity and basic properties. The polymers used for grafting include chitosan, lignin, cellulose, *etc.*^36,43^ (Fig. 4). Additionally, the mild conditions of enzymatic reactions lower the risk of thermal degradation. This modification improved more biological applications in drug delivery and tissue engineering, improving their biocompatibility. These modified grafted polymers are also helpful for environmental remedies by utilizing them in pollutant absorbents, pollutant coagulants, or making more rigid material useful for alternative plastic uses.^37–39^

### 3.4. Combined activators

Physical, chemical, and biological activators are increasingly employed in polymer grafting to enhance process efficiency and regulation. This approach utilizes multiple activators simultaneously to enhance grafting efficiency and facilitate the formation of intricate polymer structures. The integration of physical and chemical activators can optimize grafting density and dispersion, hence producing new materials for unique medical applications. Microwave-assisted radical polymerization, which integrates microwave energy with chemical activators, is an efficient technique for synthesizing graft copolymers with improved characteristics. Microwave radiation can trigger the reaction by producing radicals on the polymer backbones. When the microwave radiation is coupled with chemical activators such as ceric ammonium nitrate or AIBN they can increase grafting efficiency. The integration of microwaves with these initiators results in reduced reaction times and enhanced grafting efficiency.^40,41^ The integration of radicals and enzymes in polymer grafting presents an innovative method to enhance the grafting process. Biological and physical activators, such as UV or gamma irradiation, enhance the activation of the polymer matrix, hence facilitating efficient polymer grafting. This dual activation method combines the advantages of both enzymatic and radical processes to facilitate the synthesis of complex polymer grafts (Table 1).^42–44^ Combining the selectivity and mild conditions of enzymatic processes with the fast reaction rates enabled by radical initiators increases the synergistic effects of this method, hence improving grafting efficiency and providing better control over polymer design. An example of the combined activators used for polymer grafting is shown in Fig. 11. This method has many benefits, such as faster reaction times, high output, and the ability to keep delicate biological molecules safe. Its adaptability reaches several spheres: biomedicine, environmental science (pollutant adsorption), nanotechnology, biodegradable food packaging, and cosmetics *etc.* These activation techniques greatly increase the extent of polymer grafting for the synthesis of sophisticated materials. Here are many immobilization techniques involving “grafting from,” “grafting through,” and “grafting onto”: Tables 2 and 3.

**Table 1:** Overview of activators used in grafting

Activator type	Backbone used	Grafted chains	% grafting efficiency	Grafting condition	Application type	Ref.
High energy radiation	Ethylene vinyl acetate	Poly(butyl acrylate)	87	Gamma ray at a dose rate of 0.866 kGy h^−1^, 35 °C, 1 h	Drug carrier	45
High energy radiation	Poly(methyl methacrylate)	Polytetrafluoroethylene	97	Electron energy and beam current at 1.0 MeV and 18 mA, 35 °C, 30 min	Micropowder	46
High energy radiation	Water hyacinth fibers	Glycidyl methacrylate	56	Gamma rays at dose rate 5–30 kGy, dose rate 8 kGy h^−1^, 35 °C, 15 min	Drug delivery agent	47
Microwave irradiation	Polymethylmethacrylate	Guar gum	72	Irradiated at 900 W, 1–2min	Controlled release suspension	48
Combined activator (microwave & free radical initiator)	Gellan gum	Acrylamide	98.08	Irradiated 480 W following 1 min heating and 1 min cooling, 40 °C	Controlled release antidiabetic tablet	49
Free radical initiator	Polyacrylamide	Hydroxypropyl methyl cellulose	83.38, 71.55	Potassium persulphate as free radical initiator, 30 °C, 1 h	Controlled 5-ASA release	50
Free radical initiator	Poly(methacrylic acid)	Guar gum oleate	90	Potassium persulfate as a free radical initiator, 70 °C, 3 h	Colon-specific controlled drug delivery carrier	51
Plasma activated	Polyethylene terephthalate	Cysteamine	93	Balzers SCD050 at 35 °C, gas purity 99.997%, flow rate 0.31 s^−1^, pressure 10 Pa, electrode distance 50 mm, plasma volume 240 cm^3^, discharge power 8.3 W, 24 h	Controlled drug release	52
Enzymatic and free radical initiator	Lignin	Polyacrylamide	56.6	4 h under degassing with nitrogen	Controlled release from lignin based materials	53

**Table 2:** The immobilization of polymer on a surface using the “grafting from” and “grafting through” methods

Surface	Anchoring group	Initiating group	Polymer	Polymerization technique	Ref.
PVP-*block*-poly(4-iodostyrene) on silicon wafer	Poly(4-nitrostyrene)	Iodobenzene	Poly(3-hexylthiophene)	SI-KCTP	54
Cotton fibers	Trimethoxysilane	Iodobenzene	Polyphenylene ether	Sonogashira	55
Poly(bromostyrene) on a silicon wafer	—	Bromobenzene	Poly(9,9-bis-2-ethylhexylfluorene)	Suzuki	56
PMMA-*co*-PS-Br on SiO_2_ wafer	—	Bromobenzene	Poly(9,9-dihexyl fluorene)	Yamamoto	57
PMMA-*co*-PS-Br on SiO_2_ wafer	—	2,7-Bromo-9,9-dimethyl-9H-fluorene	Poly(9,9-dihexyl fluorene)	Yamamoto	58
Cellulose	Methanoic acid	Bromobenzene	Phenol formaldehyde resin	Yamamoto	59
Cellulose	Methanoic acid	Acetylene	Phenol formaldehyde resin	Suzuki	59
Cellulose	Methanoic acid	Acetylene	Poly(fluorene vinylene	Heck	59
Silicon wafer	Trichlorosilane	Bromobenzene	Poly[9,9-bis(2-ethylhexyl)fluorine]	Suzuki	56
Silicon and quartz wafer	Triethoxysilane	2,7-Bromo-9,9-dimethyl-9*H*-fluorene	Poly(9,9-dihexyl fluorene)	Yamamoto	58
Glass	Trimethoxysilane	Iodobenzene	Polyphenylene ether	Sonogashira	60
Silicon dioxide nanoparticles	Triethoxysilane	Iodobenzene	Polyphenylene ether	Sonogashira	61
Silicon dioxide nanoparticles	Triethoxysilane	Bromobenzene	Poly(3-hexylthiophene)	SI-KCTP	62

**Table 3:** The “grafting onto” process is used for the surface immobilization of polymer

Surface	Anchoring group	Initial group	Polymer	Chemical reaction	Ref.
Graphene	—	Azide group	Polyamide	Radical attack	63
Graphene oxide	Carbonyl chloride	Hydroxyl group	Poly(3-hexylthiophene)	Esterification	64
Graphene oxide	Carbonyl chloride	Hydroxyl group	Polythiomethylene	Esterification	65
Single walled carbon nanotubes	—	Cyclopentane	Poly(3-hexylthiophene)	Diels–Alder cycloaddition	66
Single walled carbon nanotubes	Amino group	Carboxylic acid	Poly3-octylthiophene	Amidification	67
Multi-walled carbon nanotubes	Carbonyl chloride	Hydroxyl group	Poly(3-hexylthiophene)	Esterification	68
Multi-walled carbon nanotubes	Amines	Aldehyde group	Poly(3-hexylthiophene)	Imine bond	69
TiO_2_ nanoporous	Titanium hydroxide	Carboxylic acid	Poly(3-hexylthiophene)	Direct coupling	70
TiO_2_ mesoporous	Titanium hydroxide	Carboxylic acid	Poly(3-hexylthiophene)	Direct coupling	71

## 4. Application of grafted polymer as a drug delivery system

The techniques of polymer grafting and their active roles are significantly advancing, leading to more sophisticated biomedical applications for drug delivery, bioimplants, and diagnosis, among others. The altered properties due to grafting provide more innovative, controlled, and targeted drug delivery, enhanced biocompatibility, and improved tissue adhesion. Additionally, these modifications are beneficial for stimulus-responsive drug delivery and other applications. These advancements have reduced drug leakage and side effects. Although there are many challenges in adapting this new technology for various regulatory concerns, researchers worldwide are conducting studies and finding multiple ways to treat chronic diseases.^5,72^

### 4.1. Grafted polymer on anti-cancer application

**Fig. 5:**
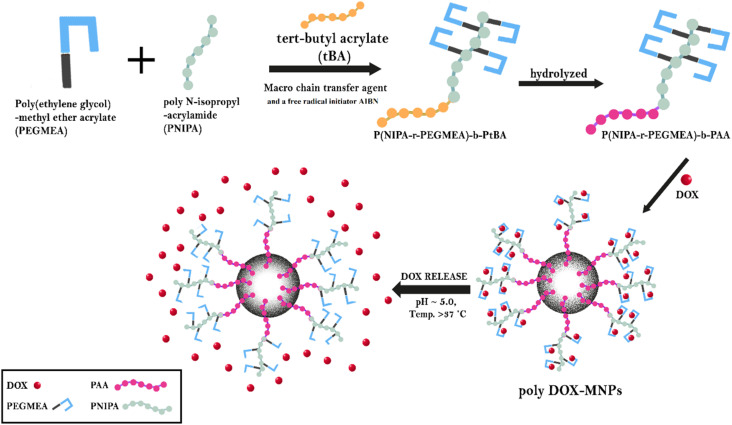
The theme of the study above forms the basis for the schematic diagram of drug release from modified polymer-coated iron oxide nanoparticles. Grafting is performed using AIBN's free radical polymerization techniques. This grafted polymer is used to deliver an anticancer drug in response to pH-responsive stimuli.

Polymer grafting offers a novel approach to delivering anticancer drugs, enabling regulated, targeted, and controlled release, while mitigating adverse effects. The bioavailability of the drug is increased by the controlled release and enhanced encapsulation of these customized polymers. The utilization of stimuli–responsive properties in grafted polymers demonstrates the development of more sophisticated anticancer treatments. These advancements have the potential to significantly enhance the outcomes of cancer treatment.^73,74^ In a study, Dutta *et al.* created pH and temperature-responsive polymer-grafted iron oxide nanoparticles by functionalizing iron oxide nanoparticles (NH_2_-magnetic nanoparticles) with “poly(*N*-isopropylacrylamide-*ran*-poly(ethylene glycol) methyl ether acrylate)-*block*-poly(acrylic acid) (P(NIPAm-PEGMEA)-*b*-PAA)” using the reversible addition–fragmentation chain-transfer (RAFT) polymerization technique with free radical initiator AIBN. The produced nanoparticles exhibited unique release behaviors influenced by environmental pH and temperature. The isoelectric point of NH_2_-magnetic nanoparticles was observed at pH 8, attributed to the presence of free primary amine groups. In contrast, polymer-coated nanoparticles demonstrated lower isoelectric points (pH 5) due to poly(acrylic acid) functionalization. *In vitro* studies showed a higher doxorubicin release rate at lysosomal pH 5.0 compared to physiological pH 7.4, with enhanced release observed at temperatures exceeding the cloud point of the grafted polymer (Fig. 5). These findings underscore the promise of polymer-grafted magnetic nanoparticles for the controlled and stimuli–responsive delivery of drugs in cancer treatment^75^

**Fig. 6:**
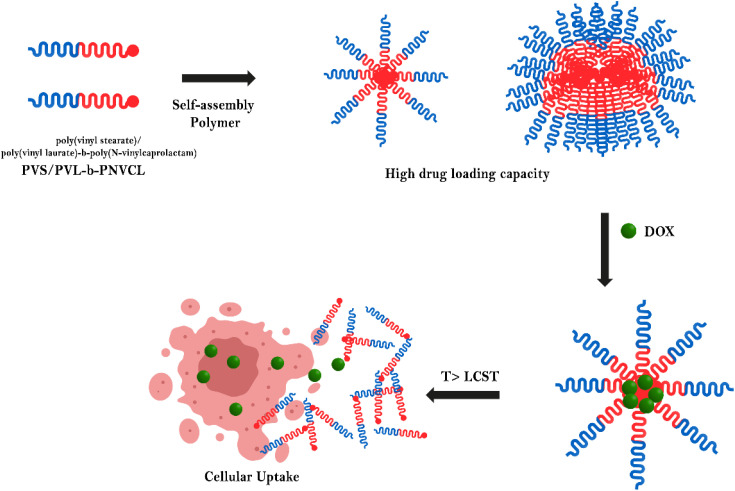
The encapsulation and release of doxorubicin from the grafted polymer PVS/PVL-*b*-PNVCL micelles are illustrated in the following schematic diagram. The microwave-assisted RAFT polymerization was employed to create these grafted block polymers. The prepared system displays the temperature-sensitive release of the drug. The scheme is derived from the theme of the study mentioned above.

Research done by Wang *et al.* investigates a novel approach to targeted cancer therapy utilizing smart, lipid-based grafted polymeric micelles (Fig. 6). To produce the grafted polymer, a series of “amphiphilic block copolymers, poly(vinyl stearate)/poly(vinyl laurate)-*b*-poly(*N*-vinylcaprolactam) (PVS/PVL-*b*-PNVCL)”, were synthesized using microwave-assisted reversible addition–fragmentation chain transfer (RAFT) polymerization. The micelles feature a lipid core for DOX drug encapsulation and a PVS/PVL-*b*-PNVCL shell, which is a polymer that responds to temperature variations. The primary innovation lies in the PVS/PVL-*b*-PNVCL shell, which retains hydration and exposes the micelle's core at reduced temperatures, facilitating drug loading. At elevated temperatures of 40–45 °C, the PVS/PVL-*b*-PNVCL shell undergoes dehydration and collapse, leading to targeted drug release at the tumor site. This temperature-sensitive transition enables controlled drug delivery, optimizing the therapeutic effect on cancer cells while reducing systemic toxicity. The lipid core facilitates the delivery of hydrophobic drugs, which are typically challenging to formulate, while the overall design enhances drug stability and prolongs circulation time within the body.^76^ This advanced delivery system holds promise for improving effectiveness and minimizing the adverse effects of anticancer medications.

Zhang *et al.* developed a stimulus-responsive polymeric nanocarrier for controlled drug delivery. The core is a copolymer, “poly(*N*-isopropylacrylamide-*co-N*,*N*-dimethylacrylamide-*b*-glycidyl methacrylate)”, created *via* a photoinduced electron transfer method using visible light and photocatalysts, ensuring precise structural control. High energy radiation activator is used for grafting. Functionalized with azobenzene (Azo) groups, these polymers self-assemble into nanoparticles in water, encapsulating 90% curcumin with sizes ranging from 220 to 600 nm. They exhibit a lower critical solution temperature (LCST) of 38.09 °C, showing heat sensitivity. The Azo groups allow UV responsiveness through *trans*–cis isomerization. Curcumin is released significantly (>80% in 3 hours) under UV light, elevated temperatures (50 °C), and alkaline pH. This nanocarrier shows promise for targeted drug delivery *via* thermal, pH, and photostimuli.^77^ Other researchers have developed a new graft copolymer, “poly(2-hydroxyethyl methacrylate-*g*-2-(dimethylamino)ethyl methacrylate)”, created by combining ATRP and RAFT polymerization processes with a free radical activator. The self-assembly behavior of this copolymer is investigated in aqueous solutions at varying pH (1, 5.5, and 7.4) and temperatures (37 °C and 42 °C), with the critical micelle concentration determined using UV-vis-NIR spectroscopy. Notably, the copolymer's self-assembly is pH and temperature-dependent. At a highly acidic pH of 1, spherical micelles form at 37 °C, while increasing the temperature to 42 °C induces a transition to cubic hexahedral micelles. Beyond characterizing self-assembly, the synthesized nanostructures are evaluated as potential doxorubicin (DOX) carriers. Drug release profiles are studied at different pH values (1.5, 5.5, and 7.4) to assess the potential for controlled drug delivery. This research offers insights into graft copolymer synthesis, stimuli–responsive self-assembly, and the development of pH-sensitive drug delivery vehicles.^78^

A research done by Hosseini Rezaei *et al.*, where they investigate novel cisplatin–polymer conjugates as potential anticancer therapeutics, focusing on enhancing drug delivery and overcoming drug resistance. In this study, carboxylated poly(2-isopropenyl-2-oxazoline) (PiPOx) was synthesized and subsequently copolymerized with methoxy poly(ethylene glycol) (mPEG) using thiol-initiated cationic ring-opening polymerization (CROP) and end-capping reactions as oxidant initiators to create micelle-forming copolymers. These copolymers were characterized using NMR, FTIR, SEC, and potentiometric titration. Cisplatin, a common anticancer drug, was then conjugated to the carboxyl groups of the copolymers at varying drug-to-polymer ratios. Cisplatin loading efficiency reached a maximum of 91% at a 1 : 1 molar ratio. The resulting “cisplatin-conjugated PiPOx-*b*-mPEG and mPEG-*g*-PiPOx” copolymers formed spherical nanoparticles with sizes of 113.3 nm and 178.8 nm, respectively, confirmed by DLS. Cytotoxicity assays in ovarian cancer cells demonstrated that the cisplatin-conjugated PiPOx-*b*-mPEG (IC50, 113 μg mL^−1^) copolymer exhibited significantly higher cytotoxicity compared to the mPEG-*g*-PiPOx (IC_50_, 232 μg mL^−1^) counterpart, suggesting its potential for improved cancer therapy, particularly in overcoming drug resistance.^79^

**Fig. 7:**
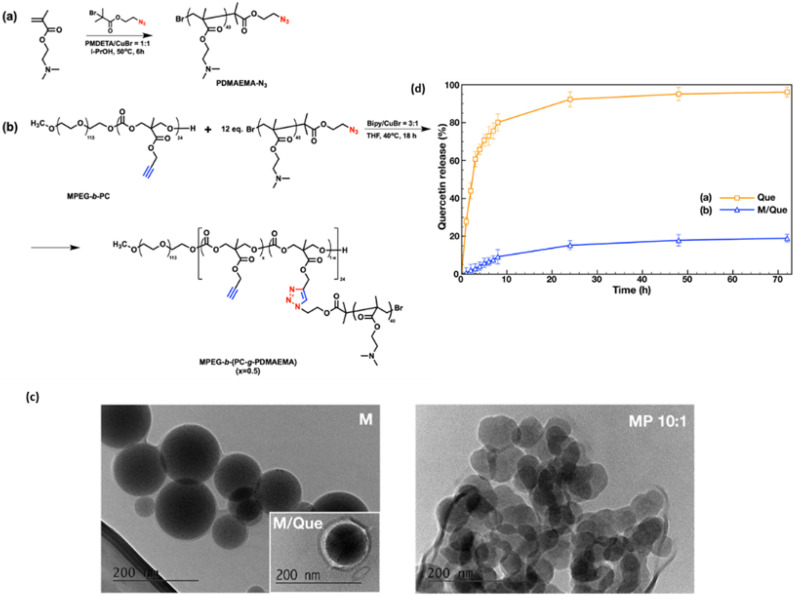
(a and b) Synthetic path of cationic amphiphilic graft copolymer MPEG-*b*-(PC-*g*-PDMAEMA); (c) morphology of quercetin-loaded graft copolymer micelles. (d) *In vitro* release profiles of quercetin from graft copolymer micelles. (Figure adapted from MDPI, license CC BY 4.0, Source^61^)

In another study Kalinova *et al.* synthesized “poly(2-(dimethylamino)ethyl methacrylate)-grafted Amphiphilic Block Copolymer Micelles” by highly efficient azide–alkyne “click” chemistry reaction illustrated in Fig. 7. The resulting copolymer self-assembled into stable cationic micelles that efficiently encapsulated both quercetin and DNA using high energy radiation activator. The MTT assay was done on a human carcinoma (HAPG2) hepatocyte cell line, which revealed that empty cationic micelles exhibit low cytotoxicity below 25 μg mL^−1^ but become highly toxic at higher concentrations due to PDMAEMA. Quercetin-loaded micelles showed enhanced cytotoxicity compared to free quercetin at low concentrations (1–5 μg mL^−1^), with comparable effects at higher doses, indicating encapsulation doesn't compromise quercetin's efficacy. Quercetin-loaded micelleplexes (10 : 1) demonstrated minimal toxicity within the DNA transfection concentration range (1–2.5 μg mL^−1^), suggesting their safety for simultaneous delivery of DNA and quercetin, although higher concentrations exhibited increased toxicity likely due to PDMAEMA. The formed micelleplexes exhibited high colloidal stability and demonstrated promising initial *in vitro* biological evaluation, suggesting their potential as safe and effective nanocarriers for the co-delivery of hydrophobic drugs and DNA.^80^

In another study, Phan *et al.* investigated the production and functionalization of polyampholytes *via* thiol–ene chemistry using microwave-assisted activation in an aqueous medium under ambient conditions. One-pot thiol–ene chemistry was employed to create durable polyampholytic alternating polymers featuring “furfuryl amine and 3-(dimethylamino)-1-propylamine as functional groups” on the surface of SWCNTs. These hybrids demonstrated consistent release behavior at pH 7.4. In contrast, a burst release at pH 5.5 indicated pH-responsive drug release, which is crucial for targeted drug delivery in the acidic tumor microenvironment. The cytotoxicity and cell viability of HeLa cells demonstrated the excellent efficiency of the anti-cancer medication.^81^

In another study, Liu *et al.* synthesized a modified natural polymer, chitosan-grafted HNTs (HNTs-*g*-CS) and investigate its potential as nano-formulation for an anticancer drug curcumin. Curcumin from HNTs-*g*-CS/Cur releases *in vitro* much more quickly in cell lysate than at pH 7.4. The HNTs-*g*-chitosan exhibits enhanced stability and hemocompatibility. The HNTs-*g*-CS exhibit enhanced hydrophobicity and surface irregularity, which are advantageous for curcumin loading.^82^ In a different study, Delorme *et al.* made PCL-*g*-Dex, a biodegradable amphiphilic graft copolymer structure based on “reverse” oligosaccharides that have hydrophobic side chains and a hydrophobic backbone using an oxidant initiator. Azido-dextran (Dex-N3) and propargylated poly(ε-caprolactone) (PCL-yne) were produced in order to further make the poly(ε-caprolactone)-*g*-dextran copolymer employing Huisgen's cyclic addition and containing the anti-cancer drug doxorubicin (DOX).^83^ At higher concentrations, free DOX caused the death of 51% of colorectal cancer cells and 55% of healthy cells, with similar curve profiles for both cell lines. Notably, drug-loaded micelles exhibited varying biological effects based on whether they were incubated with cancerous or healthy cells.

### 4.2. Anti-diabetic

**Fig. 8:**
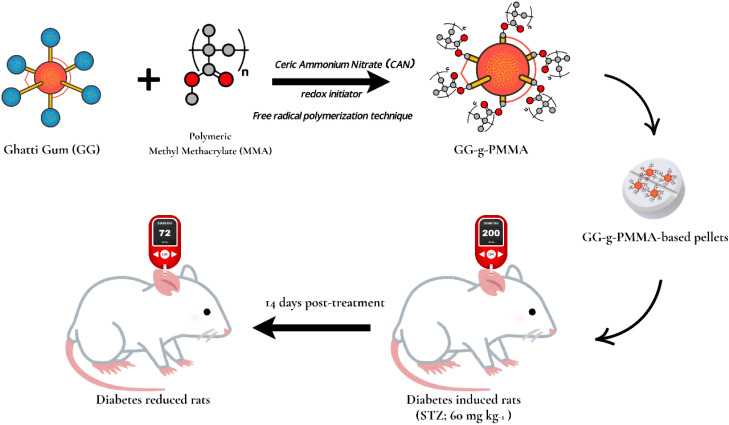
Schematic diagram of GG-*g*-PMMA-based metformin HCl pellets formulation. Here, the grafted polymer was prepared and assisted by the free radical polymerization for better drug delivery in diabetes treatment. The scheme is derived from the theme of the study mentioned above.

A promising method for enhancing the effectiveness and distribution of anti-diabetic medications is the use of polymer grafting. This method addresses the challenges in treating diabetes and has the potential to develop innovative medication delivery systems with improved treatment outcomes. In research, Bhosale *et al.* synthesized ceric ammonium nitrate (CAN) as a redox initiator to graft “ghatti gum with methyl methacrylate using the free radical polymerization technique”, thereby modifying GG-*g*-PMMA-based metformin HCl pellets (Fig. 8). *In vitro* drug release in a 0.1 N HCl solution showed that only 2.07% and 1.55% of the loaded drug were released after 2 hours. The drug was released quickly at a higher pH (pH 6.8), with 93.86 ± 2.16% after 12 h. Rats with streptozotocin-induced diabetes were used in the project to test the hypoglycemic effects of a modified GG-*g*-PMMA formulation. In comparison to the non-diabetic control group, the blood glucose levels of the diabetes control group were substantially higher, ranging from “249.87 ± 3.82 mg dL^−1^ to 263.74 ± 2.5 mg dL^−1^, compared to 68.48 ± 2.84 mg dL^−1^ to 72.52 ± 3.2 mg dL^−1^, respectively.” When compared to the diabetic control group, rats given the formulation (diabetic test group) showed a substantial decrease in blood glucose levels, primarily due to the prolonged blood glucose-lowering action of the formulation compared to the commercial formulation.^84^

Another research study, Bhosale *et al.*, synthesized polymethyl-methacrylate grafted gellan gum by the free radical polymerization technique using ceric ammonium nitrate (CAN) as a redox initiator.^85^ The synthesis of graft copolymers *via* CAN-induced free radical polymerization is a quick, reliable, and efficient method for producing pH-sensitive and sustained release polymers for targeted drug delivery systems. Adult male rats were injected intraperitoneally with nicotinamide at 110 mg kg^−1^ and streptozotocin (STZ) at 60 mg kg^−1^ to induce diabetes. Blood glucose levels were used to measure the degree of diabetes induction following 2–4 days of nicotinamide–streptozotocin therapy. A blood glucose level of more than 250 mg dL^−1^ was considered the baseline for diabetes. It displays greater anti-diabetic efficacy of the improved metformin HCl pellet formulation (batch M4) made with “Polymethylmethacrylate-*g*-gellan gum” than the commercially available Glycomet SR 500 mg tablet (diabetic standard). In another study, Gedawy *et al.* developed a silicon-grafted-alginate polymeric blend used for encapsulating metformin by a “vibrational jet flow ionotropic gelation process”.^86^ Alginate was used to homogenize polydimethylsiloxane in order to create a stable polymeric combination using oxidant initiator activation and to create microcapsules by pumping a polymeric vehicle filled with metformin *via* Buchi B-390 and into CaCl_2_. Over the course of 24 h at room temperature, the metformin-loaded novel silicon-grafted alginate platform maintained its good electrokinetic stability. After four weeks in the accelerated stability chamber (40 °C and 60% relative humidity [RH]), the microcapsules of formulations had deeper colors (brownish yellow), possibly as a result of oxidation. They had not undergone appreciable size changes. These microcapsules effectively preserve metformin content over the specified duration, with no degradation observed in any of the supplemented chromatograms.^86^

### 4.3. Wound healing

Polymer grafting enhances wound healing by modifying existing polymers with bioactive molecules or functional groups for the development of advanced wound dressings with tailored properties, such as controlled and sustained drug release, biocompatibility, higher mechanical strength, and antimicrobial activity, which better control the healing process and reduce complications. Li *et al.* employed a one-step esterification process using microwave initiator to synthesize a series of hyaluronic acid-grafted pullulan polymers with variable degrees of substitution of hyaluronic acid (HA) moieties in a study. Significantly improving the efficacy of the HA-*g*-Pu film as a wound healing material, the composition of HA is characterized by an entirely porous microstructure, a high swelling ratio, and a relatively rapid hemostatic capability^87^

. In another study, Jhong *et al.* conducted a study using atmospheric plasma-induced surface copolymerization to develop two wound-contacting membranes made of expanded “poly(tetrafluoroethylene) (ePTFE) grafted with zwitterionic poly(sulfobetaine methacrylate) (PSBMA) and hydrophilic poly(ethylene glycol) methacrylate (PEGMA)” with plasma activation. The ePTFE-*g*-PSBMA membrane accelerated wound healing more effectively than ePTFE-*g*-PEGMA or commercial dressings, demonstrating better blood-inert properties, non-bioadhesive properties, and anticoagulant activity through platelet activations in human blood. The generated wound was re-epithelialized entirely in 14 days.^88^ In another study, Duan *et al.* created a new wound dressing composite, “curcumin grafted on hyaluronic acid and modified by pullulan (Cur-HA-SPu)”. Microwave initiator is used as an activator to form the grafting. The resultant modified polymers are used to produce films, which exhibit enhanced swelling properties compared to unmodified HA films. The MTT assay was employed to conduct *in vitro* cell viability studies on L929 cells, revealing favorable biocompatibility, minimal cytotoxicity, and even promoting cell proliferation. The Cur-HA-SPu coatings also exhibited antioxidant properties and antibacterial activity against *E. coli* and *S. aureus*. In a rat wound healing model, *in vivo* studies demonstrated that Cur-HA-SPu films significantly accelerated wound healing compared to HA-SPu films or natural healing. These results indicate that Cur-HA-SPu films have the potential to accelerate wound healing and combat infection, and they are a safe and effective wound dressing material.^89^

### 4.4. Other applications

**Fig. 9:**
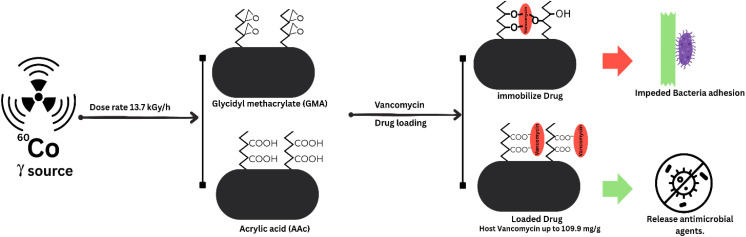
Schematic diagram of polypropylene (PP) monofilament sutures by grafting glycidyl methacrylate (GMA) and acrylic acid (AAc) using the physical activators gamma radiation. The polymer was used for the loading of vancomycin for the antibacterial application. The scheme is derived from the theme of the study mentioned above.

Like the anticancer and wound healing, the polymer grafting is also helpful in enhancing antimicrobial properties, controlling hypertension, *etc.* The grafted polymers can effectively prevent various antimicrobial infections by regulating the drug release or inhibiting microbial growth by attaching some antimicrobial agent to the polymer backbone. This approach provides a plausible solution to the challenge of antimicrobial resistance; this approach applies to various medical devices, wound dressings, and drug delivery systems. In 2024, a study done by Avval *et al.* created “acrylamide and hydroxyethylacrylate monomers grafted onto carboxymethyl starch using free radical polymerization and atom transfer radical polymerization (ATRP) techniques”. This modified grafted polymer controlled the antibiotic drug delivery at different pH levels.^90^ In another research, Vargas *et al.* created modified polypropylene (PP) monofilament sutures by “grafting glycidyl methacrylate (GMA) and acrylic acid (AAc)” using pre-irradiation with ^60^Co gamma radiation initiators (Fig. 9). After grafting, glycidyl methacrylate and acrylic acid significantly altered the suture properties, including decreased decomposition temperature and increased swellability. Glycidyl methacrylate-grafted sutures provided a smooth surface suitable for immobilizing vancomycin, reducing adhesion of *Staphylococcus aureus.* Conversely, acrylic acid-grafted sutures exhibited a rough surface and a high capacity for vancomycin loading (up to 109.9 mg g^−1^), with pH-dependent swelling behavior. Some acrylic acid-functionalized sutures demonstrated sustained antimicrobial activity. These findings highlight the potential of both glycidyl methacrylate and acrylic acid grafting to modify suture properties and create antimicrobial suture materials with controlled drug release characteristics.^91^

**Fig. 10:**
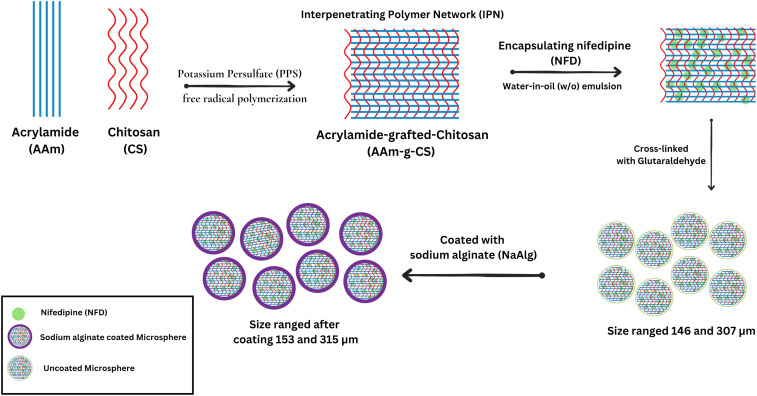
Schematic diagram on nifedipine loading in acrylamide-*g*-chitosan grafted polymer using the free radical polymerization techniques. These microspheres are used for controlled drug delivery applications. The scheme is derived from the theme of the study mentioned above.

This polymer grafting methods also has the potential to develop innovative drug delivery systems that have superior therapeutic outcomes, thereby addressing the difficulties associated with hypertension management. In oral, injectable, and implantable formulations, grafted polymers provide targeted administration and prolonged release of antihypertensive agents. In this context, Mundargi *et al.* employed a free radical initiation polymerization technique to transfer acrylamide onto xanthan gum. This technique involved the addition of ceric ammonium nitrate (CAN), as well as the anti-hypertensive drugs atenolol and carvedilol. The *F*-value (similarity factor used to compare the dissolution profiles of two drug products) of 4.64 (df = 17, *p* < 0.05) shows a significant difference in the release rates of atenolol tablets. The *F*-value, (similarity factor used to compare the dissolution profiles of two drug products) for formulations with graft copolymers containing atenolol and carvedilol was 11.95 (df = 35, *p* < 0.05). This implies that the variations in drug release rates among the formulations are significantly influenced by drug solubility. It illustrates that the release time increased in tandem with the grafting ratio of the grafted copolymer in the polyacrylamide-*g*-xanthan gum. However, there was no discernible change in the release rate between plain xanthan gum and tablet formulations containing carvedilol and graft copolymer (polyacrylamide-*g*-xanthan gum).^92^ In another study, Phadke *et al.* developed pH-sensitive microspheres for the controlled release of nifedipine. Acrylamide-*g*-chitosan copolymer was synthesized *via* free radical polymerization using a PPS initiator. Interpenetrating polymer networks (IPNs) were then formed by crosslinking acrylamide-*g*-chitosan with glutaraldehyde, followed by encapsulation of nifedipine (Fig. 10). The microspheres were further coated with sodium alginate (NaAlg) to enhance pH sensitivity. *In vitro* drug release studies revealed a pH-dependent release profile, with extended release of up to 14 hours for NaAlg-coated microspheres. This study demonstrates the successful fabrication of pH-sensitive IPN microspheres with controlled nifedipine release, showcasing their potential as promising drug delivery systems for antihypertensive agents.^93^

Contreras-García *et al.* created drug-loaded, temperature-responsive polypropylene films through (high energy activator) γ-ray pre-irradiation grafting of NIPAAm and APMA. These copolymers exhibited temperature-sensitive swelling, enhanced biocompatibility, and reduced friction. The films effectively loaded significant amounts of diclofenac and ibuprofen, with release sustained in phosphate buffer (pH 7.4, 37 °C). Films with 27% grafting released 12–13% of the drug within 30 minutes, reaching up to 41% (for diclofenac) and 70% (for ibuprofen) after 7 hours, showcasing their potential for controlled drug delivery in medical devices.^94^ Grafting can enhance the targeted distribution, controlled release, and biocompatibility of anticoagulant medications by modifying the properties of polymers. Zhu *et al.* have developed a bifunctional coronary stent characterized by enhanced biocompatibility and a diminished risk of restenosis. A zwitterionic SBMA-GMA copolymer brush was grafted through atom transfer radical polymerization (ATRP), which conferred anticoagulant properties. Additionally, DETA NONOate, identified as a nitric oxide donor, facilitated endothelialization. The coating demonstrated a controlled release of nitric oxide, robust stability, and outstanding anticoagulant activity. Both *in vitro* and animal studies substantiated improved endothelial cell proliferation, diminished cytotoxicity, and the alleviation of restenosis and thrombosis. This multifunctional stent represents a promising approach to improving long-term outcomes in cardiovascular stenting.^95^ Wang *et al.* developed a novel coronary stent surface modification to combat restenosis and thrombosis by covalently grafting a zwitterionic polymer, “poly(α-methacrylic acid-*co*-2-methyl-acryloxyethyl-phosphoryl-choline) (PMAMPC)”, and an endothelial cell-selective adhesion peptide (REDV) onto a NiTi stent using free radical initiator. This dual alteration has both anticoagulant and pro-endothelialization effects. *In vitro* investigations demonstrate good blood compatibility and increased adhesion, proliferation, and migration of endothelial cells while suppressing smooth muscle cell adhesion. The *in vivo* experiments on rats reveal excellent blood patency and quick endothelial layer development on the stent after 30 days, suggesting that this bi-functional modification technique has the potential to dramatically enhance the long-term performance of coronary stents and minimize post-implantation problems.^96^

**Fig. 11:**
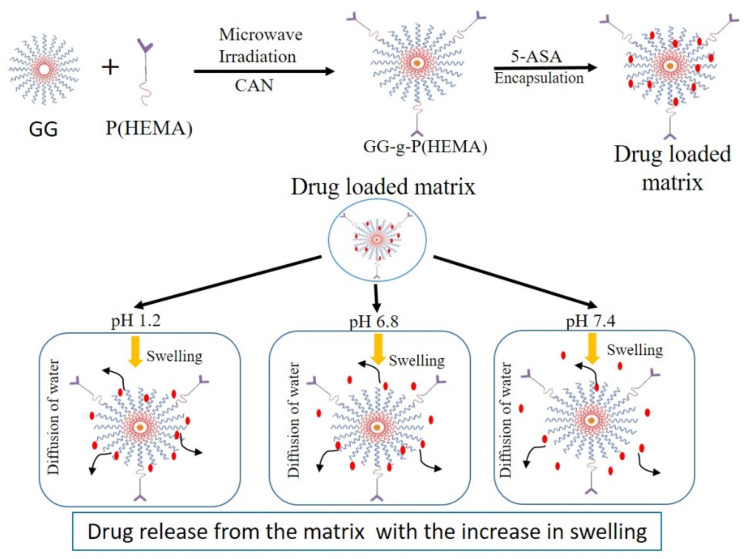
The diagram shows the drug release behavior of a grafted polymer system at various pH levels. Researchers in the study utilized combined activators for polymer grafting, thereby enhancing the material's pH-responsiveness. The grafted polymer was used for controlled drug delivery, with release kinetics adjusted according to the environmental pH.

The route of drug delivery faces specific challenges within the gastrointestinal (GI) tract. The acidic environment of the stomach, enzymatic activity in the small intestine, and varying absorption rates throughout the gastrointestinal (GI) tract can significantly influence the bioavailability and efficacy of orally administered medications. Panahi *et al.* demonstrated that the iron oxide nanoparticles were treated with 3-mercaptopropyltrimethoxysilane. Then grafted with a copolymer consisting of *N*-isopropyl acrylamide and allyl glycidyl/iminodiacetic acid by surface-initiated polymerization. The nano-sorbent exhibited a high adsorption capacity for famotidine, with a 116 mg g^−1^ value at pH 7. Furthermore, it demonstrated controlled drug release, with approximately 73% of famotidine being released in the simulated gastric fluid within one hour and 70% in the simulated intestinal fluid over 30 hours at 37 °C. These findings indicate that the developed magnetic nano-sorbent possesses significant potential as a carrier for enteric drug delivery applications.^97^ Mishra *et al.* developed a pH-sensitive drug delivery system using modified guar gum, a natural polysaccharide. The guar gum was graft-copolymerized with 2-hydroxyethyl methacrylate (HEMA) using ceric ammonium nitrate as free radical initiator, with the degree of grafting optimized by varying the concentrations of both monomers and initiator (Fig. 11). The resultant “guar gum-*g*-poly(2-hydroxyethyl methacrylate)” exhibited pH-dependent swelling behavior, with low swelling at acidic pH and high swelling at alkaline pH, thereby mimicking the conditions of the gastrointestinal tract and enabling controlled drug release. *In vitro* studies of 5-aminosalicylic acid from the developed tablets demonstrated controlled release kinetics, indicating potential for biodegradable and pH-responsive drug delivery systems derived from modified natural polymers.^98^ This material's tendency to swell makes it ideal as a drug delivery carrier.

There are some more applications of polymer grafting are given in Table 4.

**Table 4:** Different other biological application of polymer grafting

	Polymer used	Chemical reaction/techniques used	Initiator used	Surface	Size/diameter	Formulation type	Drug or API	Application	Ref.
Anti-cancer	Poly(*N*-(2-hydroxypropyl) methacrylamide di-lactate)-*co*-(*N*-(2-hydroxypropyl) methacrylamide-*co*-histidine)	Ring-opening polymerization	Stannous octoate	Poly(d,l-lactide)	200 nm	Micelles	Doxorubicin	A pH-responsive mixed micellar system utilizing graft and di-block copolymers exhibits effective drug delivery to tumors, leading to considerable tumor growth inhibition and minimal systemic toxicity	99
	Poly(l-lactide)–poly(ε-caprolactone)–poly(ethylene glycol)	Ring-opening polymerization	1,4-Butanediol	Oxidized carbon nanotubes	10–45 nm	Nanocomposites	Methotrexate	PLLA-PCL-PEG terpolymer-based nanocomposites with CNTs exhibit pH-dependent drug release characterized by Fickian diffusion as the dominant release mechanism, demonstrating their potential as effective. Carriers for methotrexate delivery	100
	l-Glutamic acid	Gamma-irradiation	—	Chitosan	Hydrogel beads 3 nm, pore size 25 μm	Hydrogel beads	Doxorubicin	pH-responsive chitosan-*g*-glutamic acid hydrogel beads exhibit excellent biocompatibility and controlled drug release properties, showcasing their potential as effective carriers for localized anti-cancer drug delivery	101
	β-Cyclodextrin	One-pot process	Carboxymethyl chitosan	Magnetic nanoparticles (Fe_3_O_4_)	38.2 nm	Nanoparticle	Prodigiosin	Enzyme-responsive, glucose-based nanocarriers with carboxymethyl chitosan show superior drug loading, targeting, and cytotoxicity against cancer cells compared to β-cyclodextrin-based carriers	102
	Methyl acrylate	Reactive extrusion	—	Graphene oxide	200–500 nm	Nanocarrier	Paclitaxel	Folic acid-functionalized graphene oxide-methyl acrylate nanocarriers for targeted delivery of paclitaxel exhibit enhanced anti-cancer activity *in vitro* and *in vivo* by attenuating mitochondrial function in breast cancer cells	103
	Poly(ethylene glycol)	Reversible deactivation radical polymerization	—	Halloysite nanotubes	20–26 nm	Nanocarrier	Quercetin	HNTs-based nanocarriers with enhanced drug loading capacity, pH-sensitive release, and excellent biocompatibility shows improved antitumor activity compared to free quercetin	104
Anti-diabetic	Catechin	Free radical polymerization	—	Inulin	4–6 μm	Nanocarrier	Inulin	Catechin-grafted inulin with enhanced thermal stability demonstrates that the graft copolymer exhibited superior α-glucosidase and α-amylase inhibitory activities compared to native inulin and catechin, suggesting its potential as a novel anti-diabetic agent	105
	Catechin	Reactive extrusion	—	Chitosan	—	Nanocarrier	Chitosan	Catechin-grafted chitosan with enhanced antioxidant and antidiabetic activities shows compared to native chitosan, demonstrating its potential as a promising biomaterial for therapeutic applications	106
	Alginate	Free radical polymerization	—	*N*-Succinylated chitosan	100–200 nm	Nanomatrix	Mangiferin	The nanoconjugate demonstrated improved hypoglycemic and hypolipidemic effects compared to free mangiferin, with a substantial reduction in blood glucose, total cholesterol, and triglycerides *in vivo*	107
Wound healing	Pullulan	Free radical polymerization	—	Methacrylate (MA) groups and β-cyclodextrin (βCD)	—	Hydrogel	Curcumin	Curcumin-loaded Pul-βCD-MA hydrogel with enhanced mechanical properties improved curcumin solubility and sustained drug release	108
	Poly(ethylene glycol)	Esterification reaction	—	Chitosan	30 μm	Film	Curcumin	The incorporation of curcumin nanoformulation into the film significantly accelerated wound closure, collagen deposition, and re-epithelialization compared to the control, demonstrating its potential as a promising biomaterial for wound healing applications	109
	Carboxymethyl guar gum	Coupling reaction	—	Ethylenediamine	—	Film	Ceftazidime	The film exhibited controlled release of ceftazidime, demonstrating biocompatibility and antimicrobial activity against *S. aureus* and *P. aeruginosa.* These findings suggest that the developed composite film possesses promising potential for biomedical applications, particularly in wound healing and tissue engineering	110
	Locust bean gum	Microwave irradiation	—	Acrylamide and acrylic acid	—	Hydrogel	C-Phycocyanin	*In vitro* and *in vivo* studies demonstrated the hydrogel's biocompatibility, antioxidant properties, and accelerated wound healing with reduced inflammation, suggesting its potential as a promising candidate for advanced wound care applications	111
Anti-microbial	(3-Acrylamidopropyl) trimethylammonium chloride	Free radical polymerization	4,4-Azobis(4-cyanovaleric acid)	SBA-15	Pore size 6–10 nm	Nanoparticle	Quaternary ammonium groups	Antimicrobial mesoporous silica materials with polymer brushes and *N*-halamine groups, demonstrate excellent antibacterial activity against *S. aureus* and *E. coli*, with potential for water filtration applications	112
	Chitosan	Free radical polymerization	—	Quercetin	—	Film	Quercetin	The antibacterial activity of Q-CS was slightly reduced compared to native chitosan, the improved antioxidant capacity of Q-CS suggests its potential for various applications in the food and healthcare sectors, such as food packaging, wound dressings, and antioxidant supplements	113
	Poly(*N*-vinyl imidazole)	Free radical polymerization	Potassium persulfate	Sodium alginate	300–600 nm	Nanoparticles	Imidazole	*N*-Vinyl imidazole-grafted sodium alginate copolymer exhibiting enhanced antimicrobial activity against Gram-positive, Gram-negative bacteria, and fungi	114
Other biological application	Poly(acryl amide)	Microwave irradiation	Ceric ammonium nitrate	Agar	—	Tablet	Mesalamine	Polyacrylamide-grafted agar *via* microwave irradiation, demonstrating its potential as a pH-sensitive carrier for controlled and colon-targeted delivery of 5-ASA.	115
	Poly(methacrylic acid)	Free radical polymerization	—	Bimodal mesoporous silicas	2–3 nm	Nanoparticles	Ibuprofen	pH-sensitive [poly(methacrylic acid)]–silica hybrid nanoparticles with controlled drug release profiles demonstrate their potential as efficient drug delivery systems	116
	Poly(vinylalcohol-*co*-ethylene)	Esterification reaction	—	Oxalic acid	7 μm × 12 μm	Hydrogel	Aspirin	pH-responsive poly(vinylalcohol-*co*-ethylene)-*g*-acetylsalicylic acid hydrogels with controlled release profiles, showing potential for targeted drug delivery in the intestine	117
	Poly(sodium styrenesulfonate)	Gamma irradiation	—	Poly(vinyl alcohol)	—	Nanoparticle	Sodium styrene sulfonate	Polyanionic chain-grafted PVA particles with enhanced anticoagulant activity demonstrate a synergistic effect of both poly(acrylic acid) and poly(sodium styrenesulfonate) grafts, exhibiting promising potential for biomedical applications	118
	Polyethylene glycol methacrylate	Atom transfer radical polymerization	2-(4-Chlorosulfonylphenyl)ethyltrimethoxysilane	316L stainless steel	20–40 nm	Nanoparticle	316L stainless steel	The surface modification of 316L stainless steel with polyethylene glycol methacrylate *via* ATRP, leading to enhanced hydrophilicity and improved anticoagulative properties	119

## 5. Conclusion and future prospect

The activator-assisted polymer grafting strategies have emerged as a promising approach for developing innovative drug delivery systems with enhanced biocompatibility, responsiveness, and specificity. This review comprehensively describes and contrasts a variety of grafting strategies, including “grafting-to,” “grafting-from,” and “grafting-through,” as well as activator techniques, including physical (*e.g.*, microwave), chemical, enzymatic, and radiation-based systems. Grafted nanocarriers, hydrogels, and implant coatings improve drug solubility, increase circulation time, and enable stimuli–responsive release for targeted therapy. Researchers widely investigate these methods in therapeutic fields such as cancer, diabetes, wound healing, and infection control. The integration of bioactive molecules with smart polymers through grafting techniques presents novel opportunities for developing advanced, targeted, patient-specific anticancer therapies.

These current technologies and advancements are effective and promising; however, certain challenges remain, notably the stability between the grafted polymer and the biological environment, as well as the influence of other excipients on the drug release profile and several additional complications. Additionally, the kinetics of drug release necessitate enhanced real-time monitoring, especially under *in vivo* conditions. Other challenges potential toxic effect from the by-products or unreacted monomers. Future research should focus on conducting more comprehensive studies to assess the effectiveness, biocompatibility, and long-term *in vivo* behaviour of various activation strategies, thereby enhancing the clinical translation of these strategies. In addition to advancing these technologies, it is necessary to strengthen polymer grafting techniques, including the application of machine learning and artificial intelligence to predict grafting efficiency, refine activator selection, and model polymer–drug interactions. Moreover, hybrid or multiple activation strategies improve reaction specificity while minimizing processing time and energy requirements. Novel materials, including metal–organic frameworks (MOFs), show promise when integrated with polymer grafting for self-adaptive, stimuli–responsive therapeutic systems. Ultimately, addressing scalability through continuous-flow reactors and green synthesis approaches is crucial for translating laboratory advances into clinical applications.

## Declaration

This review article used different language improvement software (Grammarly Premium, QuillBot Premium) to improve the grammatical errors.

## Author contributions

Mr GD and Dr AS devised and structured the study's conceptualization. Mr RB and Mr GD executed the methodology and research. Mr RB prepared all the schematic diagrams, Dr AS and Dr BD reviewed and critically revised the manuscript. All authors contributed equally to its composition and have read and consented to its submission.

## Conflicts of interest

The authors declare no conflicts of interest.

## Data Availability

No primary research results, software, or code have been included, and no new data were generated or analysed as part of this review.
